# A qualitative proteome-wide lysine crotonylation profiling of papaya (*Carica papaya* L.)

**DOI:** 10.1038/s41598-018-26676-y

**Published:** 2018-05-29

**Authors:** Kaidong Liu, Changchun Yuan, Haili Li, Kunyan Chen, Lishi Lu, Chenjia Shen, Xiaolin Zheng

**Affiliations:** 1 Life Science and Technology School, Lingnan Normal University, Zhanjiang, Guangdong 524048 China; 2 College of Food Science and Biotechnology, Zhejiang Gongshang University, Hangzhou, 310035 China; 3 College of Bioscience and Technology, Hunan Agricultural University, Changsha, Hunan 410128 China; 4 College of Life and Environmental Sciences, Hangzhou Normal University, Hangzhou, 310036 China

## Abstract

Lysine crotonylation of histone proteins is a recently-identified post-translational modification with multiple cellular functions. However, no information about lysine crotonylation of non-histone proteins in fruit cells is available. Using high-resolution LC-MS/MS coupled with highly sensitive immune-affinity antibody analysis, a global crotonylation proteome analysis of papaya fruit (*Carica papaya* L.) was performed. In total, 2,120 proteins with 5,995 lysine crotonylation sites were discovered, among which eight conserved motifs were identified. Bioinformatic analysis linked crotonylated proteins to multiple metabolic pathways, including biosynthesis of antibiotics, carbon metabolism, biosynthesis of amino acids, and glycolysis. particularly, 40 crotonylated enzymes involved in various pathways of amino acid metabolism were identified, suggesting a potential conserved function for crotonylation in the regulation of amino acid metabolism. Numerous crotonylation sites were identified in proteins involved in the hormone signaling and cell wall-related pathways. Our comprehensive crotonylation proteome indicated diverse functions for lysine crotonylation in papaya.

## Introduction

Post-translation modification (PTM) of lysine, a covalent modification involved in various biological processes, is an efficient strategy for increasing the structural and functional diversity of proteins^1,2^. To date, a large number of PTMs, including phosphorylation, ubiquitination, acetylation, and succinylation, have been identified in both eukaryotic and prokaryotic species^3–5^. PTMs alter protein functions by modulating their activity, cellular localisation, and domain structure^6^.

Lysine crotonylation (Kcr) is a recently identified PTM of histone proteins^7^. Histone crotonylation, similar to the well-studied acetylation, occurs primarily at the ε-amino group of lysine, and regulates chromatin structure and facilitates histone replacement in elongating spermatids^7^. Increasing evidence suggests histone crotonylation is not redundant to acetylation, but is essential for global transcriptional regulation in mammalian cells^8^. For example, Kcr, as an indicator of active promoters, plays an important role in the control of male germ cell differentiation^7^. Also, histone Kcr is enriched in mouse embryonic stem cells, where it is required for self-renewal^8^.

PTMs, such as succinylation, acetylation, and methylation, have been identified in both histone and non-histone proteins^4,9^. Similarly, non-histone proteins can also be modified by crotonylation. In the H1299 human lung adenocarcinoma cell line, 2696 lysine crotonylation sites have been identified in 1024 non-histone proteins^10^, while in HeLa cells, a 453 crotonylated non-histone proteins generating 1185 crotonylated peptides have been discovered^11^. In tobacco, 2044 lysine crotonylation sites distributed within 637 non-histone proteins have been identified^12^. Together, these studies indicate potentially critical and wide-ranging roles for crotonylation in multiple cellular functions.

Papaya (*Carica papaya* L.) is one of the most widely cultivated fruit crops in tropical and sub-tropical areas, and its sweet pulp of the fruit is rich in vitamins A, C, and E and carotenes^13^. Native to central America, papaya cultivation has expanded rapidly over the last few years, and both the fresh and by-products of papaya fruit are now consumed worldwide^14^. In the present study, the global lysine crotonylation proteome of papaya furit was investigated using high-resolution LC-MS/MS linked to highly sensitive immune-affinity antibody analysis^15^. To our knowledge, this was the first comprehensive analysis of lysine crotonylation in papaya, expanding our understanding of the diverse functions of this process on non-histone proteins.

## Materials and Methods

### Plant materials

Two-year-old papaya trees (*C*. *papaya* L. cv. ‘Daqing’) were planted and maintained using a standard drip irrigation and fertiliser application containing 1.425 mM NH_4_NO_3_, 0.323 mM NaH_2_PO_4_, 0.513 mM K_2_SO_4_, 0.998 mM CaCl_2_, 1.643 mM MgSO_4_, 0.009 mM MnCl_2_, 0.075 mM (NH_4_)_6_Mo_7_O_24_, 0.019 mM H_3_BO_3_, 0.155 mM CuSO_4_, 1 mM FeCl_3_, 0.070 mM citric acid, and 0.152 mM ZnSO_4_. Papaya fruits at the mature-green stage were harvested from a local commercial plantation in Zhanjiang, China. The healthy fruits of similar size, shape, and maturity were separated into three groups. Fruit samples were washed with ddH_2_O and 0.2% (w/v) hypochlorite solution for 15 min to eliminate potential microbes. The samples were treated at 25 °C with a 12 h/12 h light/dark cycle and 60–70% relative humidity.

### Protein extraction and trypsin digestion

Fruit samples were ground in liquid nitrogen, and the resultant cell powder was transferred to a 15 mL centrifuge tube. Samples were sonicated three times on ice using a high-intensity SCIENT-200F sonicator (Scientz, Ningbo, China) in lysis buffer containing 8 M urea, 10 mM DTT, 50 mM NAM, 3 μM TSA, and 1% Protease Inhibitor Cocktail (MedChen Express, Monmouth Junction, USA). Remaining debris was removed by centrifugation at 20,000 g at 4 °C for 10 min, protein was precipitated with pre-cold 15% trichloroacetic acid (TCA) for 2 h at −20 °C, the sample was centrifuged at 10,000 g at 4 °C for 10 min, and the pellet was collected and washed three times with pre-cold acetone. The protein concentration was determined using a 2-D Quant kit according to the manufacturer’s instructions (GE Healthcare, Little Chalfont, Buckinghamshire, UK). The protein sample was redissolved in buffer containing 8 M urea and 100 mM NH_4_CO_3_ (pH 8.0) for further experiments^16^.

The protein solution was reduced with 10 mM DTT for 1 h at 37 °C and alkylated with 20 mM IAA for 45 min in darkness at 25 °C. For trypsin digestion, the alkylated protein sample was first diluted with 100 mM NH_4_CO_3_, and trypsin (Promega, Madison, WI, USA) was added at a trypsin:protein mass ratio of 1: 50 for overnight-digestion, followed by a second 4 h digestion at a trypsin:protein mass ratio of 1: 100.

### HPLC fractionation and affinity enrichment

Digested peptides were fractionated by high pH reverse-phase HPLC using an Agilent 300 Extend C18 column (Agilent, Shanghai, China). Peptides were first divided into 80 fractions with a gradient of 2–60% acetonitrile in ammonium bicarbonate solution (10 mM, pH 10). Next, all peptides were combined into six fractions and vacuum dried with centrifugation.

To enrich K crotonylated (Kcro) peptides, tryptic peptides were first dissolved in NETN buffer containing 100 mM NaCl, 1 mM EDTA, 50 mM TRIS-HCl, and 0.5% NP-40 (pH 8.0), and incubated with pre-washed antibody beads (PTM Biolabs, Hangzhou, China) at 4 °C overnight with gentle shaking. Beads were washed with NETN buffer three times, and with ddH_2_O twice. Bound peptides were eluted with 0.1% TFA, and eluted fractions were combined and vacuum-dried by centrifugation. The resulting peptides were cleaned with C18 ZipTip columns (Millipore, Shanghai, China) according to the manufacturer’s instructions prior to LC-MS/MS analysis^15^.

### Quantitative proteomic analysis by LC-MS/MS

Peptides were dissolved in 0.1% formic acid and directly loaded onto an Acclain PepMap 100 reversed-phase Nano Trap Column (5 μm, 100 A, 100 μm i.d. ×1 cm; Thermo, Shanghai, China). Separation of peptides was performed using a Acclain PepMap RSLC reversed-phase analytical column (2 μm, 100 Å, 75 μm i.d. ×25 cm; Thermo, Shanghai, China). A constant flow rate of 400 nL/min solution B was maintained using an EASY-nLC 1000 UPLC system (Thermo, Shanghai, China). Gradient elution with solution B (0.1% FA in 98% ACN) was achieved by increasing the concentration from 7% to 25% over 24 min, from 25% to 40% over 8 min, increasing to 80% over 5 min, and holding at 80% for 3 min.

Resulting peptides were subjected to NSI source chromatography followed by MS/MS using a Q Exactive Plus coupled online to the UPLC instrument (Thermo, Shanghai, China). Intact peptides were detected in the Orbitrap at a high resolution of 70,000, and ion fragments were detected in the Orbitrap at a low resolution of 17,500. A data-dependent procedure, which alternated between one MS scan and 20 MS/MS scans, was applied for the top 20 precursor ions with a threshold ion count of 10,000 in the MS survey scan, with 10.0 s dynamic exclusion. The applied electrospray voltage was 2.0 kV, and automatic gain control was used to prevent overfilling of the Orbitrap. For generation of MS/MS spectra, 5E4 ions were accumulated. For MS scans, the m/z scan range was varied from 350 to 1800, and the first fixed mass was set as 100 m/z.

The mass spectrometry proteomics data have been deposited to the Proteome EXchange Consortium via the PRIDE partner repository with the dataset identifier PXD008166.

### Database search

Raw data files acquired by mass spectrometer were processed by MaxQuant and the integrated Andromeda search engine v.1.5.2.8 was searched against the *Carica papaya* L. genome database concatenated with the reverse decoy database. Trypsin/P was used as the cleavage enzyme, allowing up to 4 missing cleavages and five modifications per peptide. For precursor ions, mass error was set to 20 ppm for first stage of search and 4.5 ppm for the final search, and for fragment ions, mass error was set to 10 ppm. Carbamido-methylation on Cys was specified as a fixed modification, and three other modifications (oxidation on Met, crotonylation on Lys, and acetylation on the peptide N-terminus) were specified as variable modifications. False discovery rate (FDR) thresholds for proteins, peptides, and modification sites were set at 1%.

### Protein annotation and enrichment analysis

Gene Ontology (GO) annotation of the *C*. *papaya* proteome was performed using the UniProt-GOA database (http://www.ebi.ac.uk/GOA/). Firstly, the IDs of all identified proteins were converted to UniProt IDs and mapped onto the GO annotation proteome. Identified proteins that could not be annotated by the UniProt-GOA database were annotated by InterProScan software using the protein sequence alignment method.

The Kyoto Encyclopedia of Genes and Genomes (KEGG) database was used to annotate metabolic pathways associated with the identified proteins. The KAAS online service tools of KEGG were used to describe the KEGG annotation, and results were mapped to the KEGG pathway database by the KEGG mapper online tool.

Subcellular localisation predication was performed using WolfPsort software, and the updated version PSORT/PSORT II was used to predict eukaryotic sequences.

For GO, KEGG, and domain enrichment analysis, a two-tailed Fisher’s exact test was employed to determine the enrichment of identified proteins against all proteins in the database. Correction for multiple hypothesis testing was performed using the standard FDR control method, and a corrected *p*-value < 0.05 was considered significant^17^.

### Motif analysis

Motif-X software was used to analyse the sequence model with amino acids in specific positions in all protein sequences. Specific positions refer to modify-21-mers, with 10 amino acids upstream and 10 amino acids downstream of the specific site. All protein sequences in the database were treated as background controls.

## Results

### Proteome-wide identification of lysine crotonylation sites in *C*. *papaya* proteins

**Figure 1:**
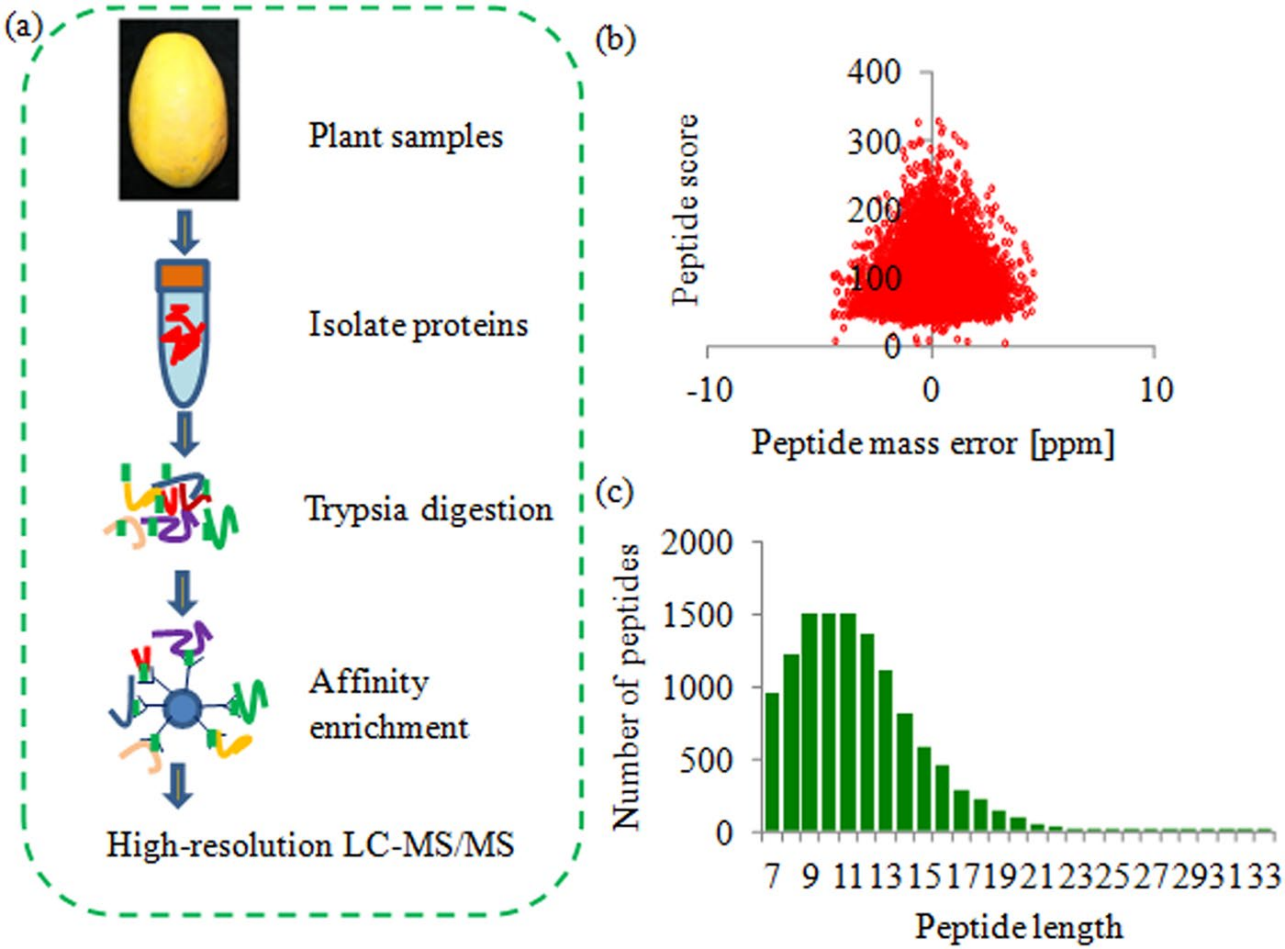
Experimental strategy and the basic information of LC-MS/MS data. (**a**) Experimental strategy for lysine crotonylation identification was showed. All crotonylated peptides were enriched by an antibody and analyzed by LC-MS/MS. (**b**) The peptides score of LC-MS/MS data. (**c**) T-distribution of crotonylated peptides based on their length.

In order to investigate the lysine crotonylation profiling of *C*. *papaya*, a combination of affinity enrichment and high-resolution LC-MS/MS was used to identify crotonylated proteins and crotonylation sites (Fig. 1a). The mass errors and lengths of all identified peptides were checked, indicating that their mass errors were ≤ 5 ppm, while the lengths of the most identified peptides varied from 7 to 17 amino acid residues (Fig. 1b,c).

In total, 5995 lysine crotonylation sites were identified in 2120 proteins. Intensive bioinformatic analyses, including GO, KEGG pathway, domain, and subcellular localization, were carried out to annotate identified lysine crotonylated proteins. Detailed information of crotonylated peptides and their matched proteins is listed in Table S1. In detail, 42.1% of all crotonylated proteins contained one crotonylation site, 21.5% of all crotonylated proteins contained two crotonylation sites, and 2.4% of all crotonylated proteins contained more than 10 crotonylation sites. Interestingly, several crotonylated proteins contained a large number of crotonylation sites. For example, a myosin-1 protein, a heat shock cognate 80 protein, and a heat shock 70 protein contained 46, 28 and 27 crotonylation sites (Table S1 and Fig. S1).

### Annotation and subcellular localization of lysine crotonylated proteins in *C*. *papaya*

Intensive bioinformatic analyses, including GO and subcellular localization, were carried out to annotate the lysine crotonylated proteins. GO functional classification of all crotonylated proteins was predicted based on three major categories: ‘Biological Process’, ‘Cell component’, and ‘Molecular Function’. Within the ‘Biological Processes’ category, the majority of crotonylated proteins were predicted to be related to metabolic processes (888 peptides), cellular processes (669 peptides), and single-organism processes (541 peptides); within the ‘Cellular Component’ category, the majority of crotonylated proteins were predicted to be associated with cell (366 peptides), macromolecular complex (225 peptides), and organelle (194 peptides); and within the ‘Molecular Function’ category, the majority of crotonylated proteins were predicted to be linked with catalytic activity (888 peptides) and binding (811 peptides) (Fig. S2).

Subcellular localization analysis showed that the majority of crotonylated proteins might be localized to the chloroplast (713 peptides), cytosol (691 peptides), nucleus (290 peptides) and mitochondria (138 peptides), respectively. Additionally, 35 proteins were predicted to be cytoskeleton-localized, and 34 were predicted to be localized to the vacuolar membrane, 19 were predicted to be localized to the endoplasmic membrane, eight were predicted to be associated with the peroxisome, and four were predicted to be localized to the Golgi apparatus (Fig. S3).

### Functional enrichment analysis

**Figure 2:**
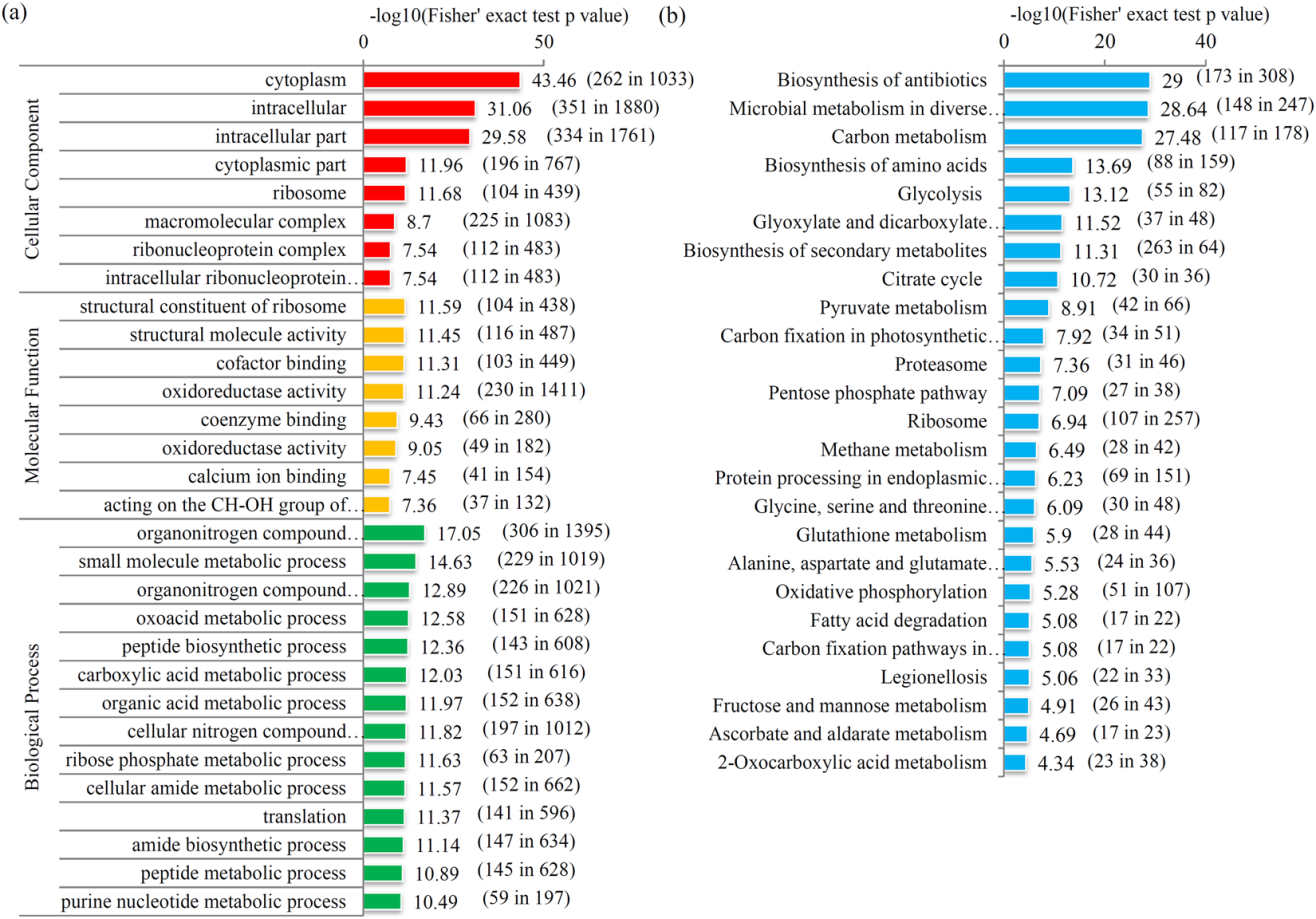
Enrichment analysis of crotonylated proteins. (**a**) GO enrichment analysis of all identified crotonylated proteins. (**b**) KEGG enrichment analysis of all identified crotonylated proteins. The negative logarithm of Fisher’s exact test *P* value was showed in X axes. The number of proteins found in each GO class and number of all proteins present in each GO class were provided in the brackets followed the scores.

To further predict the roles of crotonylated proteins, enrichment analysis was performed to classify crotonylated proteins into different groups according to their GO, KEGG, and protein domain annotations. Firstly, GO enrichment of all crotonylated proteins was predicted. For the ‘Cellular Component’ category, ‘cytoplasm’, ‘intracellular’, and ‘intracellular part’ terms were enriched; for the ‘Molecular Function’ category, the ‘structural constituent of ribosome’, ‘structural molecule activity’, ‘cofactor binding’ and ‘oxidoreductase activity’ terms were enriched, and for the ‘Biological Process’ category, ‘organ-nitrogen compound’, ‘small molecule metabolic process’ and ‘oxo-acid metabolic process’ terms were enriched (Fig. 2a).

KEGG pathway enrichment analysis showed that three KEGG pathways, including the ‘Biosynthesis of antibiotics’, ‘Microbial metabolism’, ‘Carbon metabolism’, ‘Biosynthesis of amino acids’ and ‘Glycolysis’ pathways, displayed the highest enrichment scores (Fig. 2b). For protein domain enrichment analysis, several domains, including NAD(P)-binding domain, thioredoxin-like fold, thioredoxin domain, and HSP20-like chaperone, were predicted to be significantly enriched in crotonylated proteins (Fig. S4).

### Motif analysis of lysine crotonylated peptides

**Figure 3:**
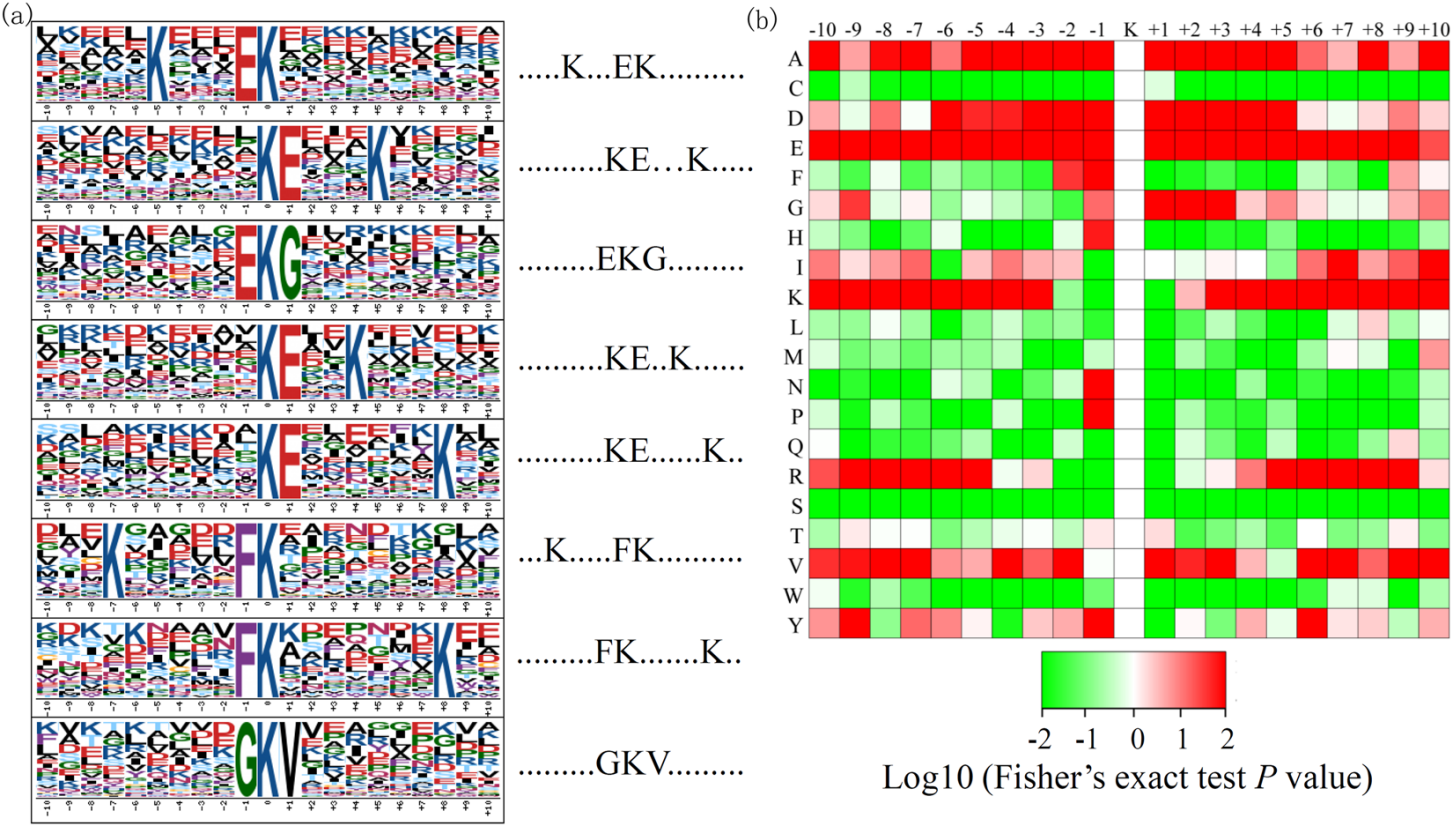
Bioinformatic analysis of lysine crotonylation sites in papaya. (**a**) Plot shows relative abundance of amino acids flanking crotonylated lysine. The relative abundance was counted and schematically represented by an intensity map. The intensity map shows enrichment of amino acids in specific positions of crotonylated lysine (10 amino acids upstream and downstream of the crotonylation site). (**b**) Probability sequence motifs of crotonylation sites consisting of 10 residues surrounding the targeted lysine residue using Motif-X. Seven significantly enriched crotonylation site motifs were identified.

To evaluate the features of the lysine crotonylated peptides, motifs in all identified crotonylated peptides were analysed. A total of eight conserved motifs (with a motif score >20) were retrieved, namely K…EKcr, KcrE…K, EKcrG, KcrE..K, KcrE……K, K…..FKcr, FKcr…….K, and GKcrV (Fig. 3a). The K…EKcr motif was present in the largest number of crotonylated peptides (144 peptides) whereas, the FKcr……K motif was the least abundant crotonylated peptides (33 peptides). The enrichment of certain amino acid residues around the Kcr in each motif was analyzed. As shown in Fig. 3b, enrichment of A, D, and E residues was observed in the −5 to −1 and +1 to +5 regions, while enrichment of K and R residues was observed in the −10 to −5 and +5 to +10 regions, respectively.

### Crotonylated proteins involved in amino acid metabolism pathways

**Figure 4:**
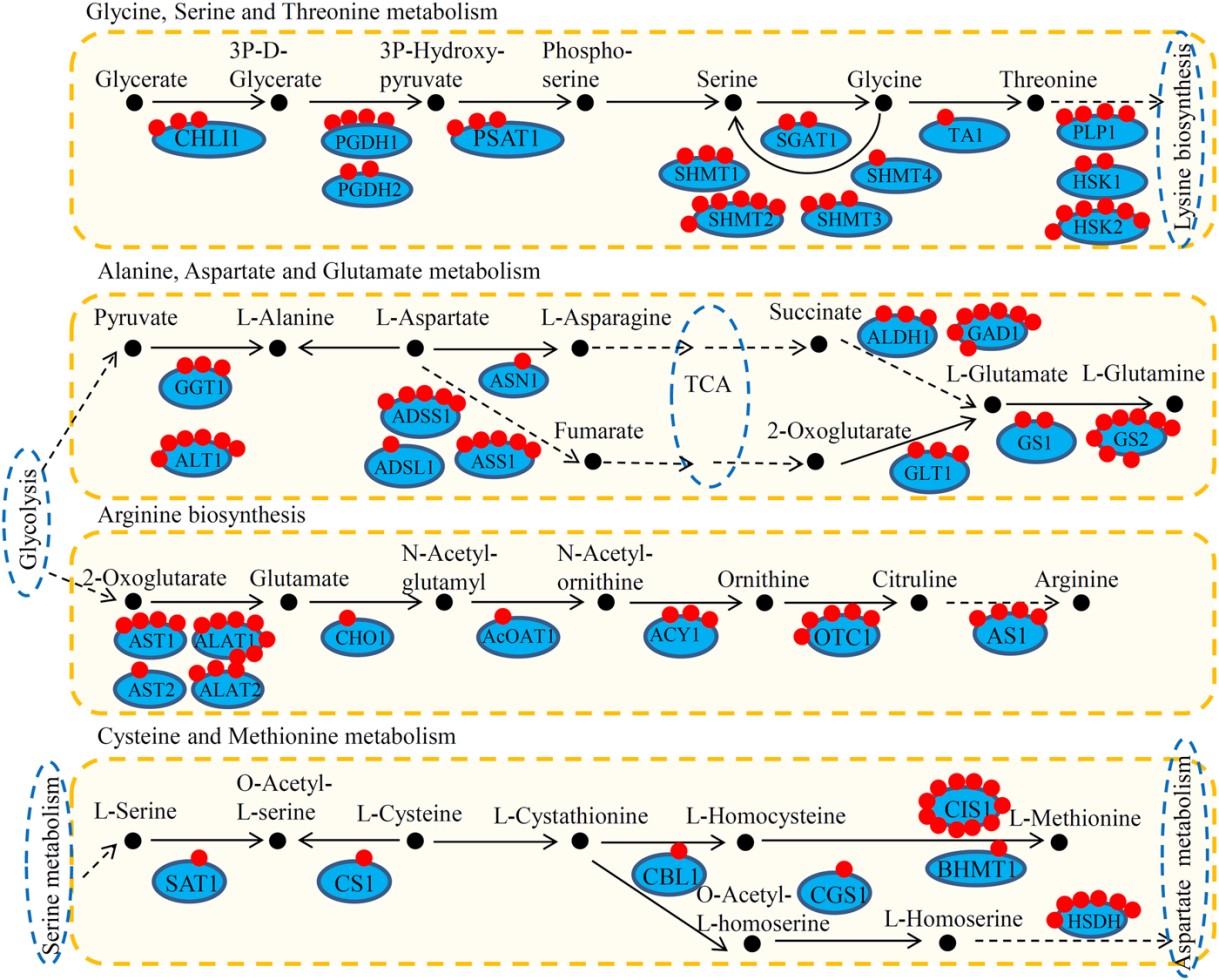
Crotonylated enzymes were involved in amino acid metabolism pathways. Red dots indicate crotonylated sites in each enzyme. Four major amino acid metabolism pathways, including Glycine, Serine and Threonine metabolism, Alanine, Aspartate and Glutamate metabolism, Arginine biosynthesis and Cysteine and methionine metabolism, were analyzed.

For glycine, serine, and threonine metabolism pathway, a total of 13 key enzymes were identified as crotonylated proteins. Among these enzymes, TA1 and SHMT4 contained one crotonylation site each, three proteins (HSK1, SGAT1 and PGDH2) contained two crotonylation sites, and SHMT2 and HSK2 contained six crotonylation sites. For alanine, aspartate, and glutamate metabolism pathway, 11 key enzymes were identified as crotonylated proteins. Among these enzymes, ASN1, GS1, GAD1, and GS2 had one, two, seven, and eight crotonylation sites, respectively. For the arginine biosynthesis pathway, 9 key enzymes were identified as crotonylated proteins. Among these enzymes, three proteins (AST2, CHO1 and AcOAT1), two proteins (ACY1 and ALAT2), and ALAT1 had one, two, and seven crotonylation sites, respectively. Moreover, for cysteine and methionine metabolism pathways, 7 key enzymes were identified as crotonylated proteins, since the proteins of SAT1, CS1, CBL1, CGS1 and BHMT1 contained one crotonylation site, while HSDH and CIS1 had six and 11 crotonylation sites, respectively (Fig. 4).

**Figure 5:**
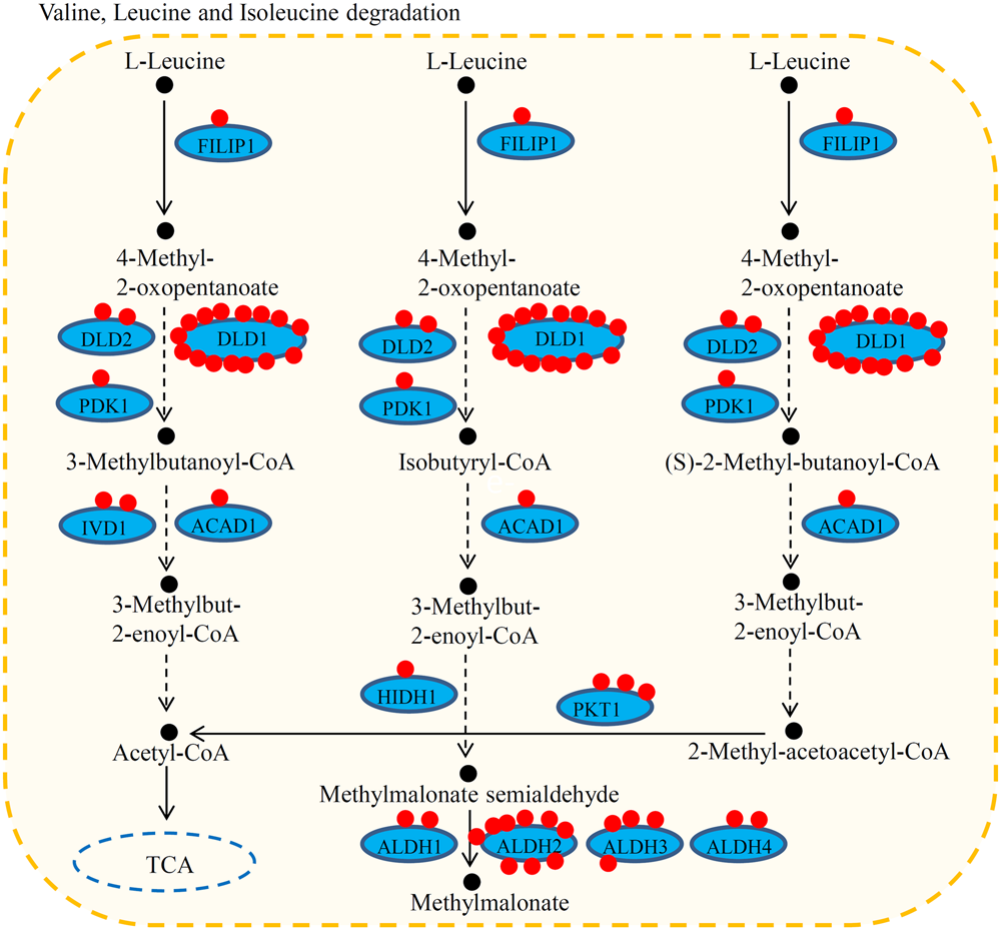
Crotonylated enzymes were involved in Valine, Leucine and Isoleucine metabolism pathway. Red dots indicate crotonylated sites in each enzyme.

For valine, leucine, and isoleucine degradation pathways, 12 key enzymes were identified as crotonylated proteins. Among these enzymes, PDK1, ACAD1, and HIDH1 contained one site each, ALDH2 and DLD1 had nine and fifteen sites respectively, and two enzymes contained a large number of crotonylation sites (Fig. 5).

### Crotonylated proteins involved in hormone signalling and cell wall-related pathways

**Figure 6:**
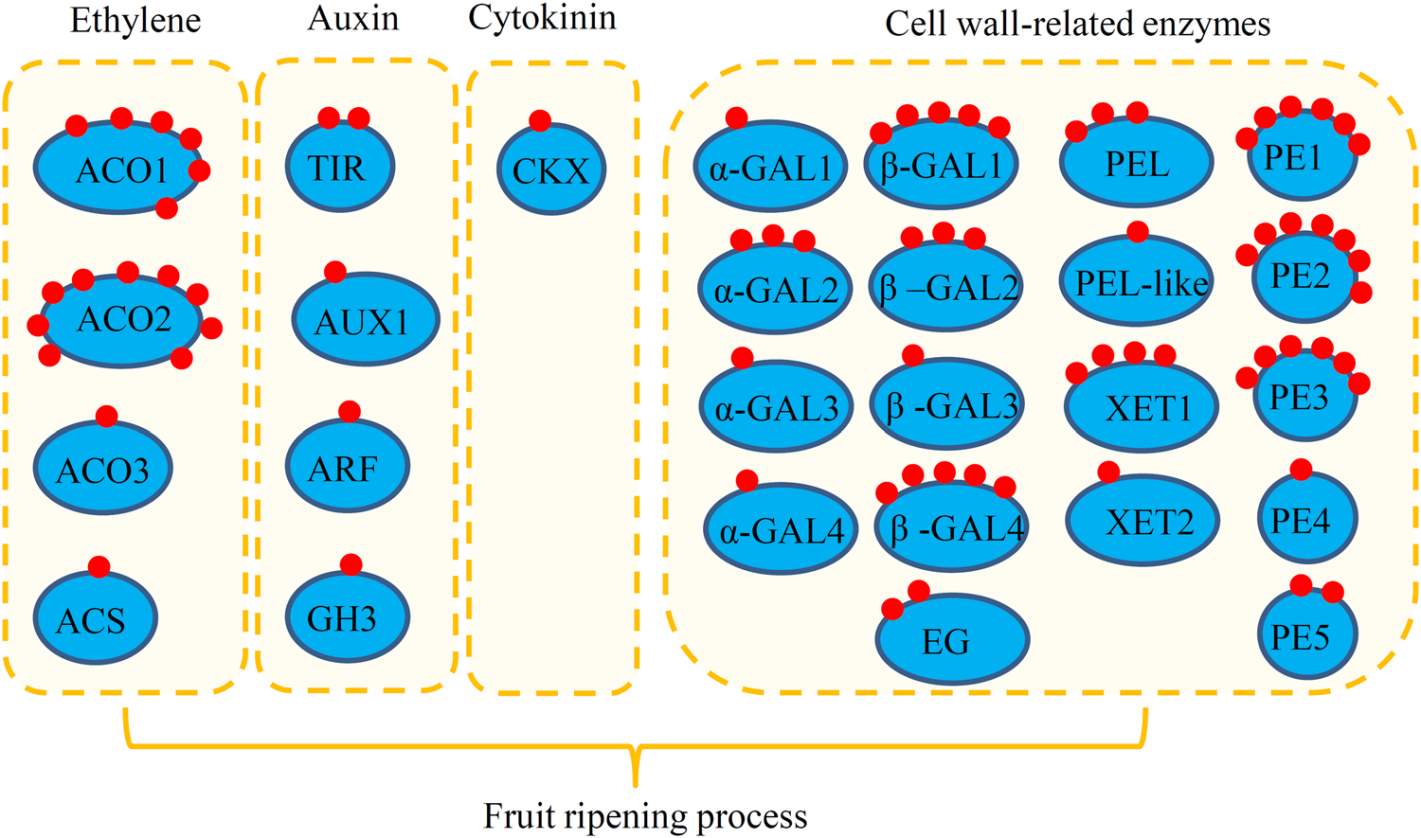
Crotonylated enzymes were involved in hormone signalling and cell wall-related pathways. Red dots indicate crotonylated sites in each enzyme.

Several hormone signaling components were identified as crotonylated proteins. For example, four ethylene-related proteins (ACO1, ACO2, ACO3, and ACS), four auxin-related proteins (TIR, AUX1, ARF, and GH3) and one cytokinin (CKX) were identified as crotonylated proteins. Moreover, a number of cell wall related proteins involved in fruit ripening were modified through crotonylation, since four α-galactosidases (α-GALs), four β-galactosidases (β-GALs), one endoglucanase (EG), one pectate lyase (PEL), one pectate lyase-like (PEL-like), two xyloglucan endotransglucosylases (XETs), and five pectin esterases (PEs) with a total of 54 crotonylation sites were identified (Fig. 6).

## Discussion

Mounting evidence suggests that PTM both histone and non-histone proteins plays a crucial role in various biological processes, including gene transcription, cellular physiology regulation, protein degradation, and cell differentiation^7,8^. Lysine crotonylation is a critical PTM that was first identified in histone proteins. As a novel and evolutionarily conserved histone PTM, lysine crotonylation is enriched on sex chromosomes in germ cell genomes^7^.

Recent studies revealed that non-histone proteins in both mammals and plants are modified by crotonylation^10,12^. A total of 453 non-histone proteins were found to be crotonylated in HeLa cells, 1024 crotonylated proteins were discovered in the H1299 human lung adenocarcinoma cell line, and 637 crotonylated proteins were identified in *Nicotiana tabacum*^10–12^. In our present study, 2120 crotonylated proteins were identified in papaya fruit, which was a larger number than that previously reported in other species, providing an opportunity to suspect the roles of crotonylation in various biological processes, such as fruit ripening.

Eight conserved motifs were identified among all crotonylated peptides in pulp of the papaya fruit, and the sequence preference for each amino acid surrounding the modified lysine was different from that identified in HeLa cells, but shared partial similarity to that identified in *Nicotiana tabacum*^11,12^. For example, motifs containing KE, EK, and FK were shared by papaya and *N*. *tabacum*, indicating a strong preference for E and K surrounding the modified lysine in plants.

Fruit ripening involves a complex series of physiological and biochemical modifications, including regulation of primary metabolism, degradation of cell walls, and changes in hormone signalling^18–20^. Papaya is a popular tropical fruit with high commercial and nutritional value^21^. Papaya fruit undergoes rapid ripening and has a short shelf-life^22^. Thus, many studies have focused on the ripening process in papaya fruit^23^.

Accumulation of amino acids, such as Glu and Asp, is contributed to improving the flavor and nutritional quality of fruit during ripening^24^. For example, L-glutamate levels increase during the ripening transition in tomato^25^. In tomato, gene expression profiling of fruits revealed that enzyme-encoding genes involved in primary carbohydrate and amino acid metabolism were up-regulated during fruit ripening^19,26^. In strawberry, the activities of amino acid metabolism pathway enzymes are varied during fruit growth and ripening^27^. In our present study, KEGG enrichment analysis showed that crotonylated proteins were enriched in several amino acid metabolism pathways, and a large number of enzymes involved in amino acid metabolism were identified as crotonylated proteins.

Plant hormones have been extensively studied to reveal their roles in the regulation of fruit development and ripening^28^. Hormones including auxin, ethylene, and zeatin collectively regulated the softening and ripening of papaya fruit by modulating cell wall softening-related enzymes^29^. In our study, nine proteins involved in hormone signalling were identified as crotonylated proteins, including four enzymes in ethylene the biosynthesis pathway, four proteins in the auxin signalling pathway, and one enzyme in the cytokinin signalling pathway. Exogenous ethylene application accelerates the ripening process of mature and premature ripening of papaya fruit^30^. Expression of ethylene-related genes such as ACO1/2/3 and ACS1 has been analysed during the ripening process in papaya fruit. These ethylene-related genes may play important roles in the abnormal softening and coloration that occur in papaya fruit upon chilling^31^. Many works have demonstrated that GH3, ARF, and Aux/IAA protein families are also involved in papaya fruit postharvest processes^20,23,32^. In our study, at least one crotonylation site in each ethylene- (ACO1/2/3 and ACS) and auxin (ARF, AUX1, TIR and GH3)-related enzyme has been identified.

During the ripening process, changes in cell wall composition contribute to a loss of firmness in papaya fruits^33^. Recently, a number of cell wall-related genes have been identified in papaya fruit^34^. In our present study, 18 cell wall-related enzymes with at least one crotonylation site were identified. Also, α-Gal, a widely-distributed enzyme in plants, acts as a hydrolase, releasing free galactose from naturally occurring galactosyl-sucrose oligosaccharides in the cell wall^35^. In ripe papaya fruits, α-Gal markedly increases pectin solubility and depolymerisation, and functions as a softening enzyme during ripening^36^. The significance of β-Gal isoforms in cell wall hydrolysis and fruit softening during ripening was evaluated over a decade ago^22^. Four crotonylated α-Gal isoforms and four crotonylated β-Gal isoforms were identified in papaya fruit (Fig. 6).

In summary, our results provided a comprehensive global lysine crotonylation proteome in papaya, a tropical and sub-tropical fruit. Our data are basic data resources for the functional validation of crotonylated proteins and a starting point for investigations into the roles of lysine crotonylation in the fruit ripening process of papaya fruit.

## Electronic supplementary material

Supplementary Information

Supplementary Dataset File
