# Supplementary material for: A qualitative proteome-wide lysine crotonylation profiling of papaya (Carica papaya L.)
Source: Sci Rep. 2018 May 29;8:8230. doi: 10.1038/s41598-018-26676-y (PMC5974297; doi:10.1038/s41598-018-26676-y)
Supplement: Supplementary file 1 — Supplementary Information [file 41598_2018_26676_MOESM1_ESM.pdf]

A qualitative proteome-wide lysine crotonylation profiling of papaya (*Carica papaya* L.)

Kaidong Liu[1,3*], Changchun Yuan[1], Haili Li[1], Kunyan Chen[1], Lishi Lu[1], Chenjia Shen[4], Xiaolin Zheng[2*]

[1] Life Science and Technology School, Lingnan Normal University, Zhanjiang, Guangdong 524048, China;

[2] College of Food Science and Biotechnology，Zhejiang Gongshang University，Hangzhou 310035, China;

[3] College of Bioscience and Technology, Hunan Agricultural University, Changsha, Hunan 410128, China;

[4]College of Life and Environmental Sciences, Hangzhou Normal University, Hangzhou, 310036, China.

*Correspondence

Kaidong Liu

Email: liukaidong2001@126.com

Xiaolin Zheng

Email: zheng9393@163.com

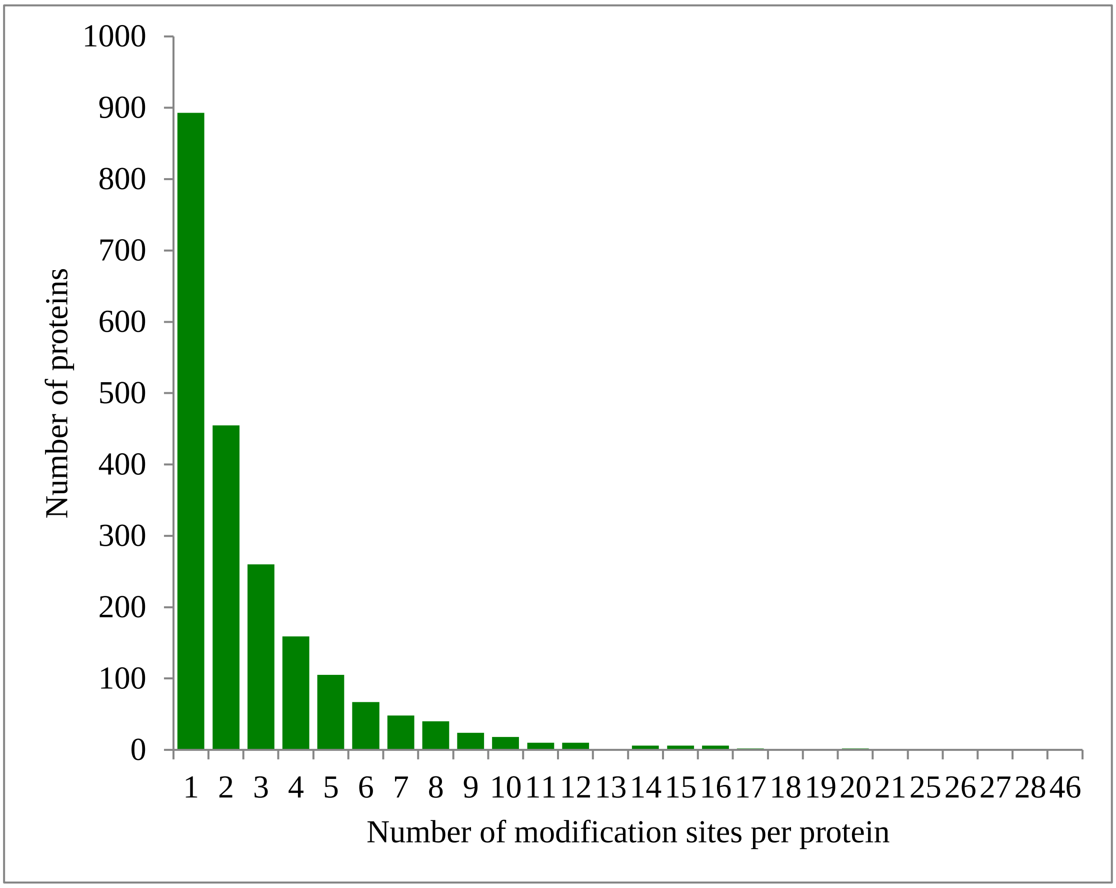

Figure S1 Number of crotonylation sites in each protein.

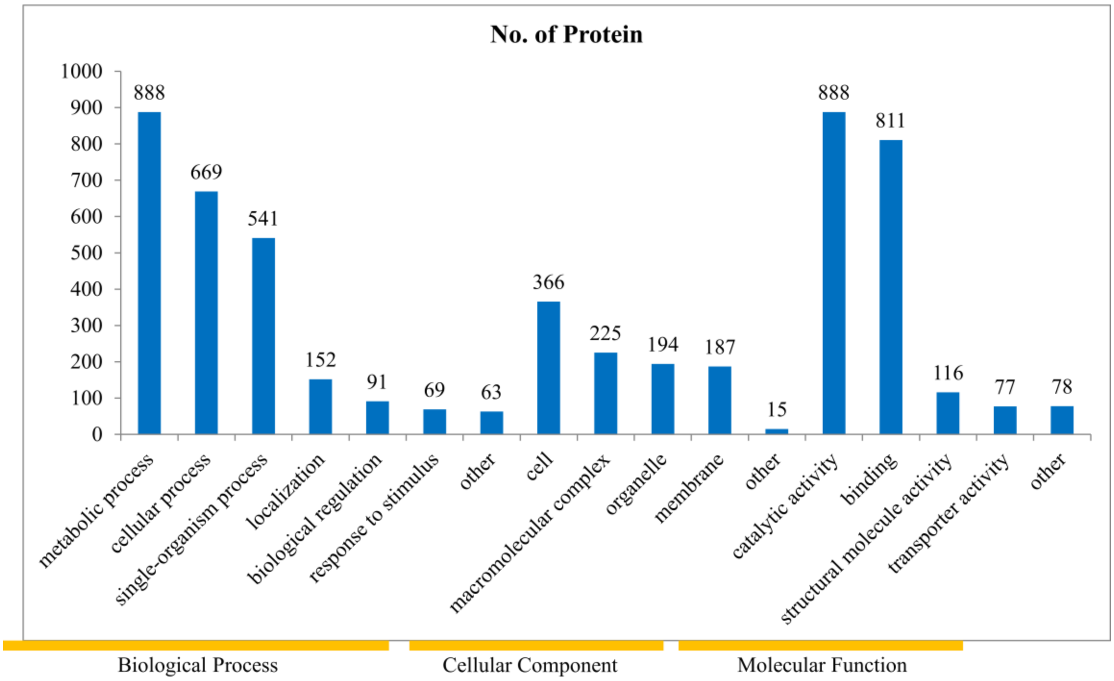

Figure S2 GO analysis of lysine crotonylated proteins in *C. papaya*.

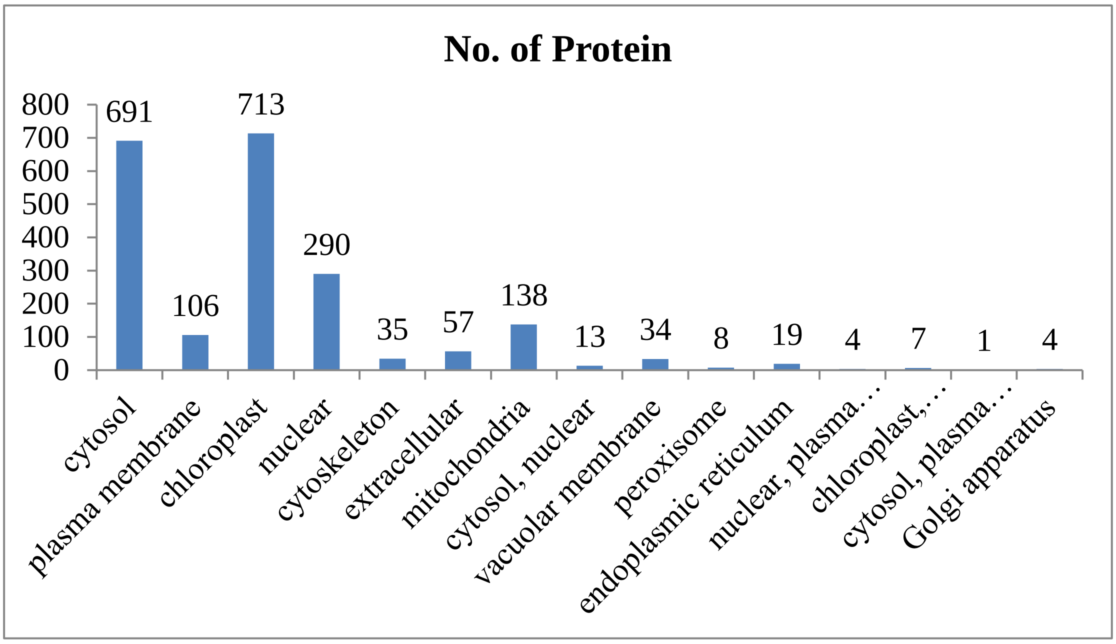

Figure S3 Subcellular localization analysis of lysine crotonylated proteins in *C. papaya*.

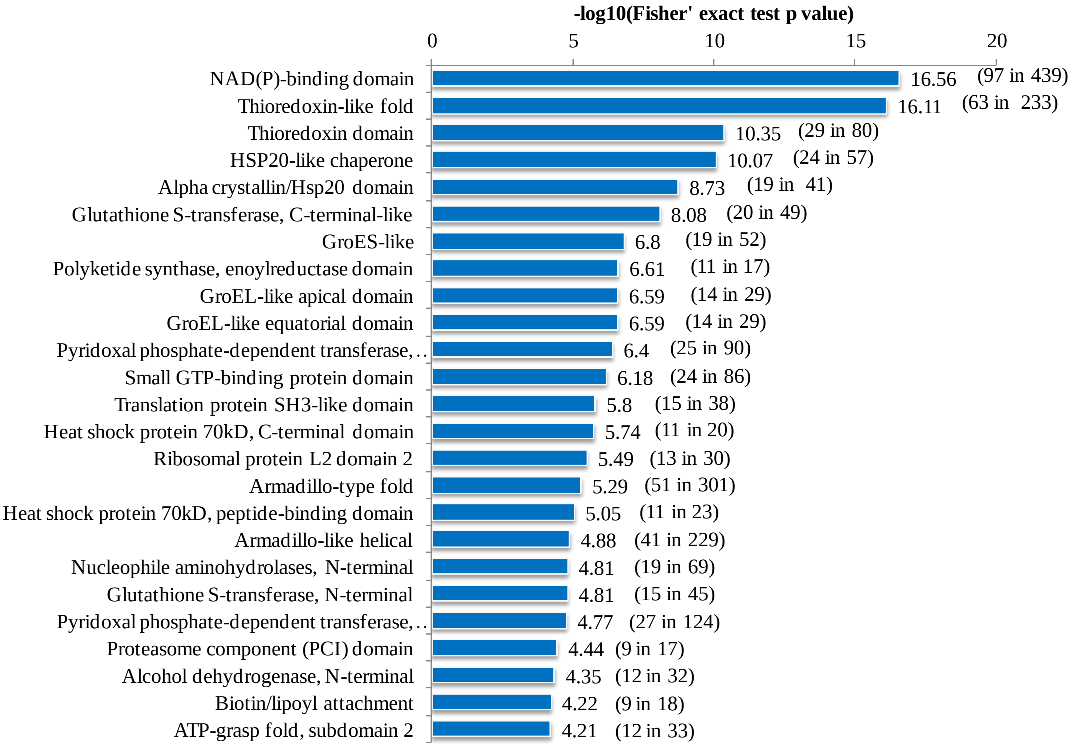

Figure S4 Domain enrichment analysis of all crotonylated proteins in *C. papaya*.
